# Camellia (*Camellia oleifera* Abel.) seed oil promotes milk fat and protein synthesis‐related gene expression in bovine mammary epithelial cells

**DOI:** 10.1002/fsn3.1326

**Published:** 2019-12-05

**Authors:** Wanqi Zhong, Jinglin Shen, Xiandong Liao, Xinlu Liu, Jing Zhang, Changhai Zhou, Yongcheng Jin

**Affiliations:** ^1^ Department of Animal Science College of Animal Science Jilin University Changchun China

**Keywords:** bovine mammary epithelial cells, camellia seed oil, casein, fatty acids, mRNA expression, protein expression

## Abstract

Camellia (*Camellia oleifera* Abel.) seed oil is a commonly used edible oil of China. In ancient Chinese literature, it is mentioned to be helpful for postpartum repair and lactation in women. Research on camellia seed oil (CO) as a feed additive for dairy cattle is less. We investigated the effect of CO on the expression of milk fat and protein syntheses‐related genes in differentiated bovine mammary epithelial cells (MAC‐T) using soybean oil (SO) as the control. The results showed that CO increased the expression of genes related to de novo synthesis of fatty acids including sterol regulatory element‐binding protein 1 (SREBP1), acetyl‐CoA carboxylase 1 (ACC), fatty acid synthase (FASN), lipoprotein lipase (LPL), and stearoyl‐CoA desaturase (SCD) (*p* < .05). Among the milk protein genes analyzed, CO increased β‐casein mRNA expression (*p* < .05) and decreased αS1‐casein mRNA expression (*p* < .05) in MAC‐T cells. CO upregulated the pathways related to milk protein synthesis with increased mRNA levels of phosphoinositide 3‐kinase (PI3K), RAC‐alpha serine/threonine‐protein kinase (AKT1), and mammalian target of rapamycin (mTOR) (*p* < .05) in MAC‐T cells. Ribosomal protein S6 kinase beta‐1 (S6K1) gene was upregulated, and eukaryotic initiation factor 4E (eIF4E) gene (*p* < .05) was downregulated with CO treatment. The mRNA expression levels of janus kinase 2 (JAK2), activator of transcription 5‐β (STAT5‐β), and E74‐like factor 5 (ELF5) were elevated in MAC‐T cells treated with CO (*p* < .05). Meanwhile, the protein expression levels of S6K1, STAT5‐β, phosphorylated mTOR (p‐mTOR), p‐S6K1, and p‐STAT5‐β increased in MAC‐T cells treated with CO (*p* < .05). In summary, CO promoted β‐casein synthesis by regulating PI3K‐mTOR‐S6K1 and JAK2‐STAT5 signaling pathways and influenced fatty acid synthesis by regulating SREBP1‐related genes in MAC‐T cells. We need to further confirm the function of CO using in vivo models.

## Graphical abstract

Camellia (*Camellia oleifera* Abel.) seed oil activated genes related to de novo synthesis of fatty acids through SERBP1 signaling pathway and promoted the synthesis of β‐casein through PI3K‐AKT‐mTOR‐S6K1 and JAK2‐STAT5 signaling pathways.

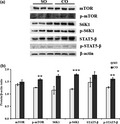

## 1 INTRODUCTION

Camellia (*Camellia oleifera* Abel.) seeds have been used in China for more than 1,000 years, and the oil extracted from seeds, named camellia seed oil (CO), is a high‐quality cooking oil (Li, Zhu, et al., 2012). Besides, CO is the most common cooking oil of the southern parts of China because camellia is widely grown there and can be stored at room temperature even in the regional climatic conditions (Ma, Ye, Rui, Chen, & Zhang, 2011; Zhong, Bedgood, Bishop, Prenzler, & Robards, 2007). CO has low polyunsaturated fatty acid content, in quantity and diversity, and preserves nutritional value (Zhong et al., 2007). It contains 80.64% oleic acid as compared to 76.16% in olive oil (Wang, Zeng, Verardo, & del Mar Contreras, 2017; Zeb, 2012). CO is considered similar to olive oil and is well known as “oriental olive oil” around the world. CO possesses various biological activities, including anti‐allergic, antioxidant, and antibacterial activities (Kim et al., 2012). Furthermore, CO is referred as a tonic for pregnant women in the “Compendium of Materia Medica,” a herbology book of medical works of the Ming Dynasty of old China (Wang et al., 2017). However, little is known on the role of CO as feed, especially in dairy cow feeding.

Manso's study has shown that dietary ingredients, especially lipid sources, such as olive oil and linseed oil, improved the fatty acid profile of milk (Manso et al., 2016). CO has higher oleic acid content; however, soybean oil (SO) is composed of only 20% oleic acid. Approximately 80% of SO produced is used in human food every year (Demorest et al., 2016; Valverde, Andjelkovic, Kundu, & Larock, 2008). SO in the diet increased milk yield and milk protein content in dairy cows and promoted lactation performance and milk fatty acid content in dairy goats (AlZahal et al., 2008; Bouattour, Casals, Albanell, Such, & Caja, 2008). However, the effect of CO, as a functional oil and rich source of fatty acids, on the nutritional composition of milk remains unclear.

Sterol regulatory element‐binding proteins (SREBPs), a family of membrane‐bound transcription factors, directly activate the expression of genes involved in the synthesis and uptake of fatty acids (Horton, Goldstein, & Brown, 2002). The in vivo study by Liang reported that oleic acid supplementation increased sterol regulatory element‐binding protein 1 (SREBP1) gene expression in bovine mammary epithelial cells (Liang et al., 2014). SREBP1 in bovine mammary gland is potentially under the control of the mammalian target of rapamycin (mTOR), a key signaling component that regulates cellular metabolism (Porstmann et al., 2008; Porstmann, Santos, Lewis, Griffiths, & Schulze, 2009). Meanwhile, mTOR regulates many components involved in milk protein synthesis. Major signaling pathways involved in milk protein synthesis are phosphoinositide 3‐kinases/RAC‐alpha serine threonine‐protein kinase‐1/mTOR (PI3K‐AKT1‐mTOR) pathway and janus kinase 2/signal transducer and activator of transcription 5 (JAK2‐STAT5) pathway (Rosen, Wyszomierski, & Hadsell, 1999; Wang & Proud, 2006). Porstmann's study indicated that PI3K‐AKT1‐mTOR regulated protein and lipid biosynthesis during mammalian cell growth in an orchestrated manner (Porstmann et al., 2009). CO is rich in fatty acids including oleic acid, which may affect both SREBP1 and mTOR‐related signaling pathways. We speculate that CO may activate fatty acid synthesis through SREBP1‐related genes and may also promote milk protein synthesis via PI3K‐AKT1‐mTOR or JAK2‐STAT5 signaling pathways.

In this study, we investigate the effect of CO supplement on the expression of genes related to milk fat and protein syntheses in bovine mammary epithelial cells (MAC‐T). This study will provide evidence for the application value of CO as a feed supplement in regulating lactation and milk protein and fat syntheses. Moreover, the study has an indirect significance for the value of feed raw materials in dairy husbandry.

## 2 MATERIALS AND METHODS

### 2.1 Source and storage of oil

Camellia seed oil (100% pure) was purchased from Yantai, Shandong province, China, and soybean oil was purchased from Shuangya mountain, Heilongjiang province, China. All oil samples were stored in airtight containers at 4°C until further use.

### 2.2 Cell culture

Bovine mammary epithelial cells (MAC‐T cells) were kindly provided by Professor Hong‐Gu Lee (Konkuk University). Approximately 3 × 10^5^ cells were seeded in 6‐well plates and maintained in DMEM/high glucose medium (HyClone; containing with 4 mM L‐glutamine, 4,500 mg/L glucose, and sodium pyruvate) supplemented with 10% fetal bovine serum (Gibco), 1% penicillin–streptomycin (HyClone), 5 μg/ml insulin (Sigma‐Aldrich), and 1 μg/ml hydrocortisone (Sigma‐Aldrich) in a 37°C incubator with an atmosphere of 5% CO_2_ and air. After attaining 100% confluency, the cells were divided into two groups as follows: control group (150 μg/ml SO as the supplement) and treatment group (150 μg/ml CO as the supplement). The cells were incubated in DMEM/high glucose differentiation medium for 4 days. During differentiation, the medium was replaced every 24 hr until harvest. The DMEM/high glucose medium (HyClone) containing 5% fetal bovine serum, 1% penicillin–streptomycin, 5 μg/ml insulin, 1 μg/ml hydrocortisone, and 5 μg/ml prolactin (Sigma‐Aldrich) was used for differentiation. Dimethylsulfoxide (DMSO, < 0.1%; Sigma‐Aldrich) was used as a vehicle to facilitate the permeation of macromolecules into live cells (Cooper, Hardin, Petersen, & Cattolico, 2010), which promotes full solubility of CO and SO in DMEM/high glucose differentiation medium. Replicates were maintained for each treatment (*n* = 6).

After 96 hr of treatment, cells were used for total RNA extraction (*n* = 3) and protein extraction (*n* = 3).

### 2.3 RNA extraction and cDNA synthesis

Cells were first washed with 1 × phosphate‐buffered saline (PBS), mixed with 1 ml of TRIzol reagent (Thermo Scientific), and scraped with a cell scraper to extract RNA. The cell lysate was passed through a pipette several times, transferred to 1.5‐mL tubes, and stored at −80°C. Total RNA was extracted from the harvested MAC‐T cells using TRIzol reagent according to the manufacturer's instructions. The concentration, purity, and integrity of the total RNA samples were assessed (optical density, OD; A260/A280 ratio) using a NanoDrop 2000 spectrophotometer (Thermo Scientific). Good quality RNA was used for cDNA synthesis.

The cDNA was synthesized from total RNA in a Life ECO gene amplification system (BIOER) using HiFiScript cDNA Synthesis Kit (CWBIO) according to the manufacturer's instructions (4 µl of dNTP mix, 2 µl of primer mix, 4 µl of 5 × RT buffer, 2 µl of DTT, 1 µl of HiFiScript, and 1 µg of RNA in a total volume of 20 µl). The cDNA was synthesized by incubating the tubes for 15 min at 42°C and cDNA amplification for 5 min in 85°C and was stored at −20°C.

### 2.4 Quantitative PCR (qPCR)

qPCR was performed on a Stratagene Mx3005P system (Agilent Technologies) using UltraSYBR mixture (CWBIO) according to the manufacturer's instructions (10 µl of 2 × UltraSYBR mixture, 0.2 µM of forward primer, 0.2 µM of reverse primer, and 10 ng of cDNA in a total volume of 20 µl). The qPCR thermal profile included an initial denaturation at 95°C for 10 min followed by quantification for 45 cycles (95°C for 10 s, annealing at 60°C for 30 s, and extension at 72°C for 32 s). Next, the dissolution curve was analyzed (95°C for 15 s, 60°C for 1 min, 95°C for 15 s, 60°C for 15 s). The relative change in gene expression was analyzed using 2‐^ΔΔCT^ method. β‐actin was used as the reference gene. Primer sequences are listed in Table 1.

**Table 1:** *Bos taurus* primers used for real‐time PCR

Gene name	Accession number	Primers sequence	Product size, bp
β‐actin	NM_173979.3	F: 5’‐CTCTTCCAGCCTTCCTTCCT−3’ R:5’‐GCAGTGATCTCTTTCTGC−3’	178
α_S1_‐casein	NM_181029.2	F: 5’‐ACTGAGGATCAAGCCATGGAAG−3’ R:5’‐GAATGTGCTTCTGCTCAACACT−3’	100
β‐casein	XM_010806178.2	F: 5’‐ACCAGCCTCTTCCTCCAACT−3’ R:5’‐GCCTGAATGGGCATATCTCT−3’	124
JAK2	XM_005209981.4	F: 5’‐CAAGACCAGATGGATGCCCAG−3’ R:5’‐ACTCGAACTGCTAGGTCTCTGA−3’	103
STAT5	NM_174617.4	F: 5’‐GAGAACACCCGCAATGATTAC−3’ R:5’‐TCACCGACTCTGCTCCACG−3’	151
ELF5	NM_001024569.1	F: 5’‐CATCCGCTCACAAGGTTACTC−3’ R:5’‐CTCGCACAAATTCCCATAGAT−3’	170
PI3K	NM_001206047.1	F: 5’‐GTCTGGACCTTCGGATGCTAC−3’ R:5’‐TAAACTCCTCAATGGCTCGGT−3’	213
AKT1	NM_173986.2	F: 5’‐GCACAAGCGAGGTGAGTACAT−3’ R:5’‐GCCACGGAGAAGTTGTTGAG−3’	138
mTOR	XM_002694043.6	F: 5’‐CGAAGAACCAATTATACCCGC−3’ R:5’‐CATAGCAACCTCAAAGCAGTCC−3’	153
S6K1	NM_205816.1	F: 5’‐AATGCTGCTTCTCGTCTTGGA−3’ R:5’‐CAGTTCTTCCCAGTTAATATGTCT−3’	90
eIF4E	NM_174310.3	F: 5’‐CCCGCCTACAGAAGAAGAGA−3’ R:5’‐CAGTATCAAACTTAGAGATCAATCG−3’	164
ACC	NM_174224.2	F: 5’‐GGAGACAAACAGGGACCATTAC−3’ R:5’‐GTGGAAGGAATGCTTGGGAG−3’	187
FASN	NM_001012669.1	F: 5’‐GACCTGGGAGGAGTGTAAGC−3’ R:5’‐GCGATAGCGTCCATGAAGTA−3’	198
SCD	NM_173959.4	F: 5’‐CCACGTTCTTCATTGATTGC−3’ R:5’‐CAGCCACTCTTGTAGCTTTCC−3’	121
LPL	NM_001075120.1	F: 5’‐TCACTTCAACCACAGCAGCA−3’ R:5’‐GATGACGTTGGAGTCCGGTT−3’	127
PPARγ	NM_181024.2	F: 5’‐TTCCGTTCCCAAGAGCTGAC−3’ R:5’‐TGGGGATACAGGCTCCACTT−3’	98
SREBP1	NM_001113302.1	F: 5’‐CGCTCTTCCATCAATGACA−3’ R:5’‐TTCAGCGATTTGCTTTTGTG−3’	188

### 2.5 Western blot analysis

Cells were washed twice with ice‐cold 1 × PBS and mixed with 0.5 ml of RIPA lysis buffer (R0278; Sigma‐Aldrich) containing 1 × protease inhibitor cocktail (M250, Amresco Biochemicals and Life science) to extract protein. The mixture was incubated for 30 min at 4°C, and then, the cells were scraped off with precooled cell scrapers, transferred to 1.5‐mL tubes, vortexed, and centrifuged at 12,000 × g for 30 min at 4°C. The supernatant was collected, packed, and then stored at −80°C for Western blot analysis. Transfer the samples from −80°C to 4°C for freeze–thaw. The protein concentration was measured using bicinchoninic acid (BCA) protein assay (Pierce). According to the amount of protein required, different volumes of 2X SDS Loading Sample Buffer(0.5 mM Tirs/HCl pH 6.8, 4% SDS, 20% glycerol, 1% β‐mercaptoethanol, and 1% bromophenol blue）were mixed, respectively, vortexed for 20 s, and centrifuged at 1,000 *g* at 4°C for 1 min. Immediately heat the mixed samples at 100°C for 5 min. After heating, let stand at room temperature, vortexed for 20 s, and centrifuged at 1,000 *g* at 4°C for 1 min. Use 1.5‐mm‐thick electrophoretic glass plate, the sample protein (10 μg) was separated by SDS‐PAGE (10%, w/v) and transferred to nitrocellulose membrane (Millipore Corp). The membrane was blocked for 3.5 hr with 5% skim milk in TBST buffer (a mixture of tris‐buffered saline and Tween 20; pH 7.6; 20 mM Tris‐HCl, 137 mM NaCl, and 0.01% Tween 20), incubated overnight at 4°C with the primary antibody (rabbit anti‐β‐actin polyclonal antibody (AF7018, 1:2000, 43 kDa; Affinity Biosciences), rabbit anti‐mTOR polyclonal antibody (bs‐1992R, 1:2000, 289 kDa; Bioss), rabbit antiphospho‐mTOR (Ser2448) polyclonal antibody (bs‐3494R, 1:2000, 289kDa; Bioss), rabbit antibovine STAT5 polyclonal antibody (bs‐1142R, 1:2000, 90kDa; Bioss), rabbit antiphospho‐STAT5a (Ser726) polyclonal antibody (bs‐5619R, 1:2000, 91kDa; Bioss), rabbit anti‐S6K1 polyclonal antibody (bs‐6370R, 1:2000, 70kDa; Bioss), or rabbit antiphospho‐S6K1 (Ser417) polyclonal antibody (bs‐5668R, 1:2000, 58kDa; Bioss), washed 4 times with 1 × TBST, incubated for 4 hr at 4°C with goat anti‐rabbit IgG‐HRP antibody (sc‐2004, 1:2000; Sant Cruz Biotechnology), further incubated with ECL Western blotting substrate (Thermo Scientific) for 5 s, and visualized using a chemiluminescence imaging system (Tanon). The protein band intensity was quantified using ImageJ 1.52a software (National Institutes of Health). The protein level was normalized by comparing the signal with β‐actin on the same membrane.

### 2.6 Statistical analysis

Data were analyzed using SPSS 17.0 (SPSS Inc.) and expressed as mean ± standard error. The treatment effects were evaluated using independent *t* test.

## 3 RESULTS

### 3.1 Camellia seed oil promotes expression of SREBP1 gene and de novo fatty acid synthesis‐related genes in MAC‐T cells

**Figure 1:**
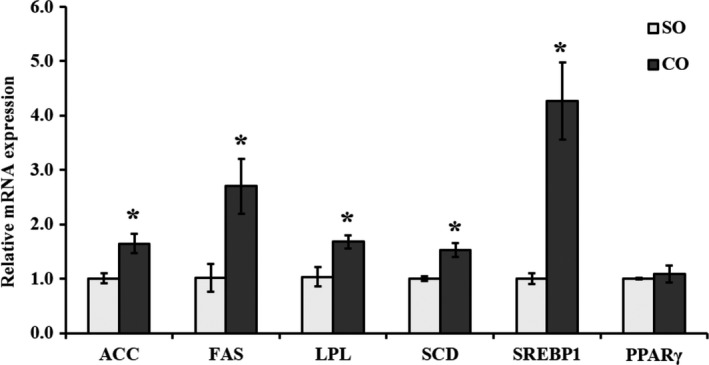
mRNA expression of fatty acid synthesis‐related genes in MAC‐T cells treated with camellia seed oil. Data are mean ± *SEM* (*n* = 3); * indicates significant difference compared with the control (MAC‐T cells treated with SO) at *p* < .05. SO: soybean oil, CO:camellia seed oil

Addition of CO significantly increased SREBP1 mRNA expression in MAC‐T cells compared with the control (*p* < .05; Figure 1). The mRNA levels of genes involved in de novo synthesis of milk fatty acids including acetyl‐CoA carboxylase (ACC) (*p* < .05), fatty acid synthase (FASN) (*p* < .05), lipoprotein lipase (LPL) (*p* < .05), and stearoyl‐CoA desaturase (SCD) (*p* < .05) were upregulated in MAC‐T cells treated with CO compared with control (Figure 1). However, the mRNA level of peroxisome proliferator‐activated receptor gamma (PPARγ) (*p* > .05) remained unchanged (Figure 1).

### 3.2 Camellia seed oil promotes β‐casein gene expression and reduces αS1‐casein gene expression in MAC‐T cells

**Figure 2:**
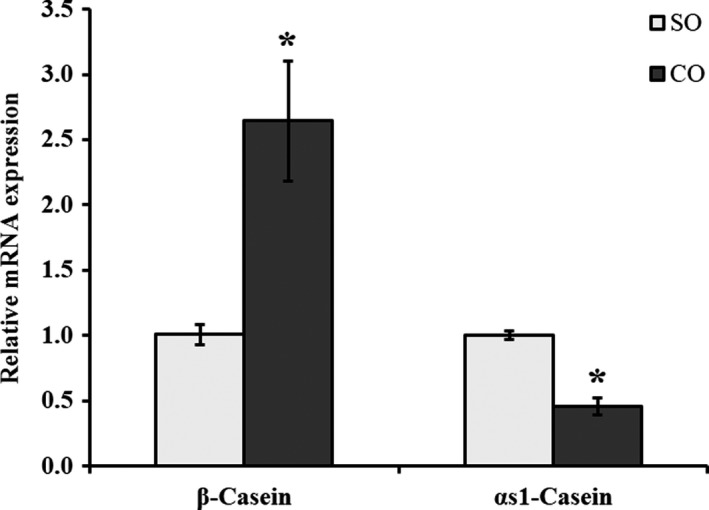
mRNA expression of caseins in MAC‐T cells treated with camellia seed oil. Data are mean ± *SEM* (*n* = 3); * indicates significant difference compared with the control (MAC‐T cells treated with SO) at *p* < .05. SO: soybean oil, CO:camellia seed oil

We examined the effect of CO on mRNA expression of β‐casein and αS1‐casein genes in MAC‐T cells through qPCR analysis. β‐casein mRNA expression in MAC‐T cells increased significantly with CO treatment compared with control (*p* < .05; Figure 2). However, αS1‐casein mRNA expression was downregulated in MAC‐T cells treated with CO compared with control (*p* < .05; Figure 2).

### 3.3 Camellia seed oil activates PI3K‐AKT‐mTOR and JAK2‐STAT5 signaling pathways in MAC‐T cells

**Figure 3:**
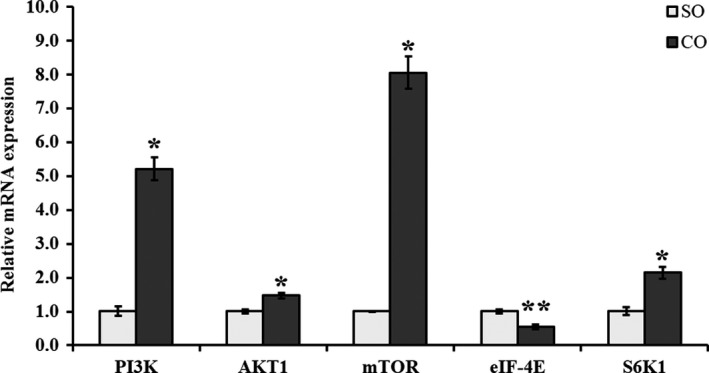
mRNA expression of PI3K‐AKT‐mTOR signaling pathway‐related genes in MAC‐T cells treated with camellia seed oil. Data are mean ± *SEM* (*n* = 3); * and ** indicate significant differences compared with the control (MAC‐T cells treated with SO) at *p* < .05 and *p* < .01, respectively. SO: soybean oil, CO:camellia seed oil

The mTOR mRNA was upregulated in MAC‐T cells treated with CO (*p* < .05; Figure 3). Western blot analysis revealed that the protein levels of mTOR (*p* < .05) and phosphorylated (p‐)‐mTOR (*p* = .002) were high compared with control (Figure 5). The results of qPCR were confirmed by Western blot analysis. In addition, we observed that the upstream genes PI3K and AKT1 of the mTOR were significantly upregulated in CO treatment (*p* < .05; Figure 3). We further examined the downstream genes, ribosomal protein S6 kinase 1 (S6K1) and eukaryotic initiation factor 4E (eIF4E) of mTOR. S6K1 mRNA was significantly upregulated in CO treatment (*p* < .05; Figure 3); however, eIF4E mRNA was significantly downregulated in CO treatment (*p* = .005; Figure 3). Further, Western blot analysis revealed that the protein levels of S6K1 (*p* < .05) and p‐S6K1 (*p* = .0002) were high in MAC‐T cells treated with CO compared with control (Figure 5).

**Figure 4:**
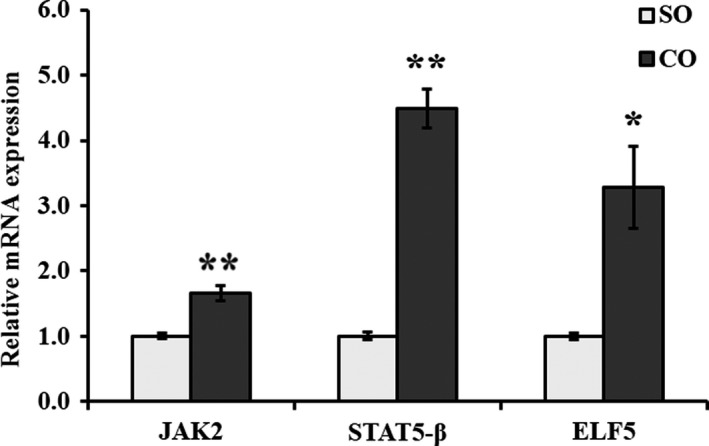
mRNA expression of JAK2‐STAT5 signaling pathway‐related genes in MAC‐T cells treated with camellia seed oil. Data are mean ± *SEM* (*n* = 3); * and ** indicate significant differences compared with the control (MAC‐T cells treated with SO) at *p* < .05 and *p* < .01, respectively. SO: soybean oil, CO:camellia seed oil

**Figure 5:**
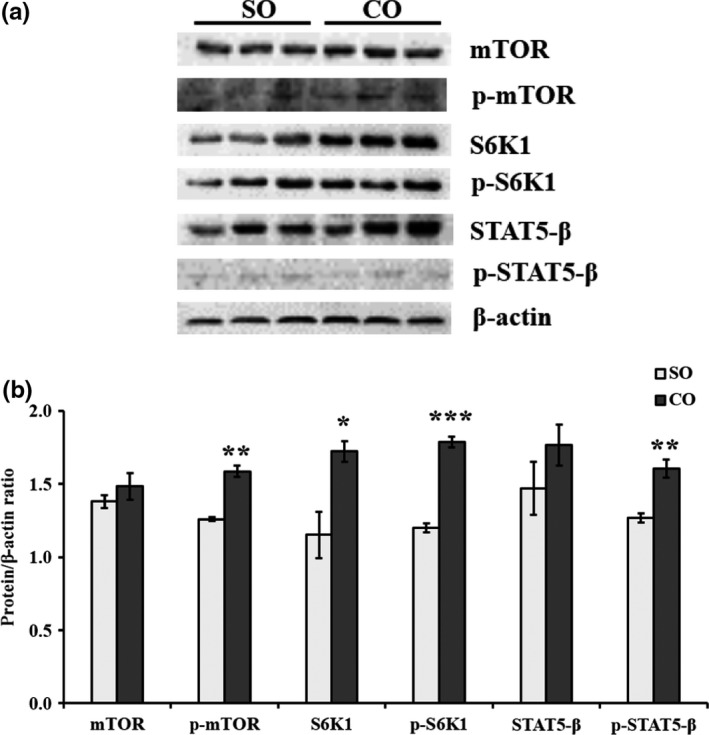
Protein levels of mTOR, phosphorylated (p‐)‐mTOR, S6K1, p‐S6K1, STAT5‐β, and p‐STAT5‐β in MAC‐T cells treated with camellia seed oil. Representative Western blot images for mTOR, p‐mTOR, S6K1, p‐S6K1, STAT5‐β, and p‐STAT5‐β in MAC‐T cells are shown in (a). Protein to β‐actin ratios are shown in (b). Data are mean ± *SEM* (*n* = 3); * and *** indicate significant differences compared with the control (MAC‐T cells treated with SO) at *p* < .05 and *p* < .001, respectively. SO: soybean oil, CO:camellia seed oil

Additionally, we detected the expression of genes of JAK2‐STAT5 signaling pathway. The mRNA levels of JAK2 (*p* = .005) and E74‐like factor 5 (ELF5) (*p* < .05) were significantly elevated in MAC‐T cells treated with CO compared with control (Figure 4). STAT5‐β mRNA was also significantly upregulated in MAC‐T cells treated with CO compared with control (*p* = .003; Figure 4). The protein levels of STAT5‐β (*p* < .05) and p‐STAT5‐β (*p* = .008) were significantly high in MAC‐T cells treated with CO (Figure 5).

## 4 DISCUSSION

CO is a nutrient‐rich organic woody vegetable oil that contains plenty of unsaturated fatty acids (UFA), such as linoleic acid and oleic acid (Su, Shih, & Lin, 2014). Meanwhile, SO is the most commonly used edible oil and is high in polyunsaturated fatty acids (Demorest et al., 2016). The UFA content in CO can reach up to 90%, which is perhaps the highest amount reported so far among vegetable oils (Cheng, Lu, & Yen, 2015; Feás et al., 2013). Loor's study reported an increase in milk fat and protein contents with linseed oil supplementation in grazing dairy cows’ diet (Loor, Ferlay, Ollier, Moreau, & Chilliard, 2005). Bouattour's study showed that SO added to the diet of dairy goats promoted lactation performance and increased milk fatty acid content (Bouattour et al., 2008). However, few studies have so far investigated the effect of CO on the expression of genes regulating milk fat or protein synthesis.

SREBP1 plays a central role in regulating milk fat biosynthesis, and upregulation of SREBP1 expression can promote milk fat synthesis and secretion (Bionaz & Loor, 2008). In our experiment, the addition of CO promoted the expression of SREBP1 gene and induced the expression of FASN, ACC, and LPL genes. These results are consistent with the findings of Bionaz. The expression of genes involved in the de novo synthesis of fatty acids such as FASN, a complex homodimeric enzyme (Roy et al., 2006), ACC, a highly regulated enzyme in the fatty acid synthesis pathway (Peterson, Matitashvili, & Bauman, 2003), and LPL, a gene involved in fatty acid transport (Bionaz & Loor, 2008; Peterson et al., 2003). Wakil and Gao's study reported the role of LPL in fat deposition (Gao et al., 2019; Wakil & Abu‐Elheiga, 2009). In our experiment, LPL gene was significantly upregulated in cells treated with CO, which may promote the absorption of fatty acids in MAC‐T cells. FASN is a multifunctional protein that plays a central role in mammals in de novo lipogenesis (Roy et al., 2006). In this study, FASN and ACC genes were significantly upregulated. These findings indicate that de novo fatty acid synthesis‐related genes are activated by CO in MAC‐T cells.

Expression of SCD, the most abundant gene measured, is important during milk fat synthesis (Peterson et al., 2003). SCD, a SREBP‐responsive gene, is the key enzyme in the synthesis of monounsaturated fatty acids in the mammary gland (Rincon et al., 2012). Li's study showed that supplementation of safflower oil or linseed oil increased the expression of ACC, LPL, and SCD genes in the mammary tissues of lactating goats (Li, Yan, Lee, Choi, & Song, 2012). Our experiments in MAC‐T cells also showed that SCD gene was significantly upregulated with CO treatment probably because CO is rich in oleic acid. PPARγ plays a dominant role in adipose cell differentiation (Tontonoz & Spiegelman, 2008). However, there was no change in PPARγ expression with CO treatment. It can be concluded that the addition of CO can promote de novo synthesis of fatty acids and further affect milk fat synthesis by regulating SREBP gene.

Casein is a protein unique to milk, and it accounts for 80% of total milk proteins. The casein family includes α‐, β‐, and k‐caseins (Bai et al., 2008; Mohanty, Mohapatra, Misra, & Sahu, 2016). AlZahal's study showed that SO linearly increased milk yield and milk protein yield in lactating dairy cows (AlZahal et al., 2008). In this study, we observed that β‐casein was upregulated (*p* < .05) and αS1‐casein was downregulated (*p* < .05) with CO treatment. These findings, together with the earlier evidence, suggest that the addition of nutrients including CO can promote casein synthesis in MAC‐T cells. To further elucidate the effect of CO on casein synthesis in MAC‐T cells, we examined the expression of related genes and proteins of two classical signaling pathways of milk protein synthesis.

mTOR is an important regulator of milk protein synthesis (Yang, Kennelly, & Baracos, 2000). An earlier study by Osorio, Lohakare, and Bionaz (2016) showed that the overexpression of SREBP1 increased the expression of mTOR in bovine mammary epithelial cells (Osorio et al., 2016). In our study, the mRNA expression level of mTOR was upregulated (*p* < .05) and the protein or phosphorylated protein expression level of mTOR was also upregulated with CO treatment (*p* < .05) compared with SO. From our study, it can also be concluded that the upregulation of SREBP gene may regulate the expression of mTOR gene in MAC‐T cells, which is consistent with Osorio's study. Burgos's study also showed that nutrients (amino acids or glucose and acetate) and lactogenic hormones (hydrocortisone, insulin, and prolactin) through mTOR signaling pathway modulated milk protein synthesis (Burgos, Dai, & Cant, 2010).

Additionally, in our experiment, PI3K and AKT1 upstream genes of the mTOR signaling pathway were also upregulated (*p* < .05) with CO treatment. The protein synthesis appears to be regulated by downstream genes of the mTOR pathway in all tissues found in mammals and the expression of milk protein synthesis‐related genes such as S6K1 and eIF4E (Rius et al., 2010; Wang & Proud, 2006). mTOR promotes protein synthesis mostly via phosphorylating effectors, such as S6K1, and by energizing the mTOR signaling cascade. Our experiments revealed that the mRNA expression level of eIF4E was downregulated; however, the mRNA expression level (*p* < .05) and the protein or phosphorylated protein expression level of S6K1 were upregulated with CO treatment (*p* < .05) compared with SO. This finally affects the cellular mechanism of protein synthesis through changes in eIF4E and S6K1, which play major roles in the translation of ribosomal mRNA sequences (Hayashi et al., 2009; Saxton & Sabatini, 2017). Zhang et al. (2014) also observed that methionine increased the mRNA levels of β‐casein, AKT1, mTOR, and S6K1 in bovine mammary epithelial cells. These results indicated that CO, as a feed supplement, promoted β‐casein gene expression via PI3K‐AKT‐mTOR‐S6K1 signaling pathway.

The mTOR signaling pathway remains active throughout the lactation cycle and interacts with JAK2‐STAT5 signaling pathway to regulate the synthesis of lactation‐related and hormone‐induced proteins (Rius et al., 2010). JAK2‐STAT5 signaling pathway is a recently discovered signal transduction pathway mediated by cytokines, which plays an important role in cell proliferation, differentiation, and immune regulation. In addition, JAK2‐STAT5 signaling pathway is a hotspot in milk protein synthesis research (Bionaz, & Loor, 2011). In this study, mRNA expression levels of JAK2 and STAT5‐β were also upregulated (*p* < .05). Moreover, the protein or phosphorylated protein expression level of STAT5‐β was upregulated (*p* < .05) with CO treatment compared with SO. Harris's study showed that ELF5 upregulated the activity of STAT5 in mammary epithelial cells, regulated the differentiation of mammary epithelial cells, and promoted lactation (Harris et al., 2006). In this study, mRNA expression level of ELF5 was upregulated (*p* < .05). STAT5 regulates the synthesis of lactoprotein by promoting the secretion of prolactin and other growth factors. As the downstream gene of STAT5, the core factor in JAK2‐STAT5 pathway, transcription factor ELF5 assists STAT5 and regulates the synthesis of lactoprotein, affects the expression of key genes in lactation process and increases the activity of STAT5 during lactation (Bionaz & Loor, 2008). Yang's study showed that Met‐Met promoted casein gene expression in cultured mammary gland explants by activating JAK2‐STAT5 and mTOR signaling pathways by enhancing intracellular substrate availability (Yang et al., 2015). Nan et al. (2014) reported that lysine and methionine promoted mRNA levels of casein genes by increasing the mRNA levels of JAK2, STAT5‐β, ELF5, and mTOR and by reducing the mRNA levels of eIF4E in bovine mammary epithelial cells. Therefore, it can be concluded that adding nutrients such as CO can promote JAK2‐STAT5 and mTOR signaling pathways.

This study showed that the increased β‐casein expression in MAC‐T cells treated with CO was associated with JAK2‐STAT5 signaling pathway. mTOR and JAK2‐STAT5 signaling pathways interact to regulate lactation‐related factors and hormone‐to‐protein synthesis (Wang et al., 2014). Signaling pathways of milk fat and protein syntheses were significantly upregulated in MAC‐T cells treated with CO. Thus, it can be concluded that the addition of CO can promote PI3K‐AKT‐mTOR and JAK2‐STAT5 signaling pathways at mRNA and protein (including phosphorylated) levels.

## 5 CONCLUSIONS

In conclusion, CO activated genes related to de novo synthesis of fatty acids through SERBP1 signaling pathway and promoted the synthesis of β‐casein through PI3K‐AKT‐mTOR‐S6K1 and JAK2‐STAT5 signaling pathways. We can conclude that the addition of CO can increase the beneficial nutrients in milk and therefore may be a potential feed supplement for dairy cattle. We need to further evaluate the feed value of CO through feeding experiments in ruminants.

## CONFLICT OF INTEREST

The authors declare that they do not have any conflict of interest.

## ETHICAL APPROVAL

This study does not involve any human or animal testing.
